# Profile of patients attended as psychiatric emergencies at a university general hospital

**DOI:** 10.1590/1516-3180.2013.1316598

**Published:** 2013-12-01

**Authors:** Vitoria Mantoan Padilha, Carolina Silva Said Schettini, Amilton Santos, Renata Cruz Soares Azevedo

**Affiliations:** I MD. Psychiatrist, Department of Medical Psychology and Psychiatry, School of Medical Sciences, Universidade Estadual de Campinas (Unicamp), Campinas, São Paulo, Brazil.; II Undergraduate Student, Department of Medical Psychology and Psychiatry, School of Medical Sciences, Universidade Estadual de Campinas (Unicamp), Campinas, São Paulo, Brazil.; III MD, MSc. Psychiatrist, Department of Medical Psychology and Psychiatry, School of Medical Sciences, Universidade Estadual de Campinas (Unicamp), Campinas, São Paulo, Brazil.; IV MD, PhD. Psychiatrist and Professor, Department of Medical Psychology and Psychiatry, School of Medical Sciences, Universidade Estadual de Campinas (Unicamp), Campinas, São Paulo, Brazil.

**Keywords:** Emergency services, psychiatric, Emergency medical services, Emergency service, hospital, Psychiatric department, hospital, Mental health, Serviços de emergência psiquiátrica, Serviços médicos de emergência, Serviço hospitalar de emergência, Unidade hospitalar de psiquiatria, Saúde mental

## Abstract

**CONTEXT AND OBJECTIVE:**

The prevalence of psychiatric conditions in clinical settings is high, particularly in emergency services. This is a challenge for healthcare professionals and an essential element in the functioning of the mental health network. The objective here was to describe the sociodemographic and clinical profile and the practices among patients treated psychiatrically in the Emergency Unit.

**DESIGN AND SETTING:**

Descriptive and quantitative study, conducted at Hospital das Clínicas (HC), Universidade Estadual de Campinas (Unicamp).

**METHODS:**

Sociodemographic data, reasons for attendance, diagnostic hypotheses and practices were analyzed.

**RESULTS:**

Psychiatric staff attended 1,835 cases over the study period, corresponding to 1465 patients. The patients were predominantly women (53.7%) and white (79.6%); their mean age was 37 years and 41.3% lived with their parents. The commonest reasons for attendance were depressive symptoms (28.1%), agitation (23.6%) and problems with psychoactive substances (19.5%). The commonest diagnoses were psychoactive substance-related disorders (23%) and depressive disorders (18.5%). 31.6% of the patients were referred to healthcare centers and 29.2% to specialized outpatient clinics, while 8.2% were hospitalized.

**CONCLUSIONS:**

This study emphasizes that it is important for professionals working in emergency service to have information about the patients' profile and the main reasons that lead them to seek psychiatric care, and to establish a diagnosis that will allow proper management at the emergency service and case referral.

## Abstract (pt)

**CONTEXTO E OBJETIVO:**

A prevalência de quadros psiquiátricos em contextos clínicos é elevada, particularmente em serviços de emergência, representando um desafio para os profissionais da saúde e um elemento essencial no funcionamento da rede de saúde mental. Objetivou-se descrever o perfil sócio-demográfico, clínico e condutas para pacientes atendidos pela psiquiatria na Unidade de Emergência.

**TIPO DE ESTUDO E LOCAL:**

Estudo descritivo e quantitativo, conduzido no Hospital das Clínicas (HC), Universidade Estadual de Campinas (Unicamp).

**MÉTODOS:**

Foram analisados dados sócio-demográficos, motivos de atendimento, hipóteses diagnósticas e condutas.

**RESULTADOS:**

Houve 1.835 atendimentos pela Psiquiatria no período, correspondendo a 1.465 pacientes. Encontrou-se predomínio de mulheres (53,7%), idade média de 37 anos, maioria branca (79,6%); 41,3% moravam com os pais. Os motivos mais frequentes de atendimento foram sintomas depressivos (28,1%), agitação (23,6%) e problemas com substâncias psicoativas (SPA) (19,5%). Os diagnósticos mais frequentes foram transtornos relacionados a SPA (23%) e transtornos depressivos (18,5%). 31,6% dos pacientes foram encaminhados para centros de saúde, 29,2% para ambulatórios especializados e 8,2% foram internados.

**CONCLUSÃO:**

O estudo reforça a importância de que profissionais que atuem em serviços de emergência tenham informações sobre o perfil, os principais motivos que levam os pacientes a procurar atendimento psiquiátrico, além do estabelecimento de uma hipótese diagnóstica que permita uma adequada conduta no serviço e o seu encaminhamento.

## INTRODUCTION

Mental disorders have high rates of prevalence in the population and represent a significant demand for healthcare services.1 Among the medical conditions that require attention, psychiatric emergencies can be highlighted. These can be defined as any situation of a psychiatric nature in which there is a significant risk of death or serious injury to the patient or to others, thus requiring immediate therapeutic intervention.^2^

In England in 1967, Bridges^3^ had already reported that it was important to set up psychiatric emergency services (PES) for better care of patients with mental disorders, claiming that general practitioners (GPs) had difficulty in managing these patients, especially due to the stigma surrounding mental illness. In the United States, PES emerged during the 1960s as one of the services considered essential for attending to local communities' mental health.^4^ From 1992 to 2001, in the United States, there was a substantial increase in the number of visits to emergency units due to psychiatric conditions (from 17.1 to 23.6 per 1000 inhabitants).^5^

In Brazil, particularly since the changes to mental health policies that began in the mid-1980s,^6^ care for patients with mental disorders has started to be based on outpatient services and PES have taken a prominent role in the care network.^7^

PES have an important role in indicating the treatment for each case, playing a role in screening new cases, placing patients in the available service network, assessing and treating acute behavioral changes and associated medical conditions, and providing help in hospitalizations, especially in non-operating periods at other services.^4^ ^,^ ^8^

In view of this scenario, in which attendance of psychiatric emergencies is both a challenge for healthcare professionals and an essential tool for proper functioning of integrated mental healthcare services, it is essential to understand the characteristics of the population attended, in order to optimize care and treatment flow.

## OBJECTIVE

To describe the sociodemographic and clinical profile of the population attended by the psychiatric staff at an emergency referral unit (ERU).

## METHODS

### Type of study

This was a descriptive and quantitative study that evaluated data from the medical files of all patients seen by the psychiatric staff over a 12-month period starting in May 2010.

### Local

The ERU of Hospital das Clínicas (HC), Campinas State University (Universidade Estadual de Campinas, Unicamp) provides high complexity healthcare services for the metropolitan area of the city of Campinas, covering a population of around 5,000,000 inhabitants. The main objective of the ERU is to provide specialized care for urgent and emergency medical cases, such that patients presenting the most serious conditions have priority of medical care. Referral of cases of most medical specialties within the coverage area, to the ERU, is done primarily through telephone contact from public rescue systems (mainly the Mobile Emergency Attendance Service, Serviço de Atendimento Móvel de Urgência, SAMU) or from the vacancy regulators at the Regional Healthcare Administration Centers (of Campinas and neighboring municipalities). Prior to the medical consultation, patients undergo screening by nurses, and those with potentially more serious conditions are given priority of attendance. There are also patients who seek the service spontaneously and, once again, the criterion of case severity, as evaluated through the nurses' screening, is respected in determining the order of attendance.

The case volume at the ERU is 200 adult patients per day, on average. Patients under 14 years of age are evaluated separately, by the Pediatric Emergency Service of HC/Unicamp. The psychiatric care is administered by doctors in the first, second and third years of the medical residency program in Psychiatry, accompanied by medical students and monitored on site by a supervisory psychiatrist, every day of the week, 24 hours a day.

### Sample

All patients of both genders who sought psychiatric care during the study period were included. Recurrent appointments for the same patient during the study period were excluded, such that only the first visit of each patient were taken into consideration. Patients who had more than one course of care during the period (repeaters) were analyzed separately, in order to evaluate whether they constituted a group with particular characteristics.

It is important to report that the sample was composed exclusively of patients who sought psychiatric medical care. Patients who appeared in the records of referrals requested by other specialties at the ERU were not included.

For all the patients seen by psychiatric staff at the ERU, the following data were collected from the medical records: name, registration number, gender, city of origin, age, marital status, race, education level, profession, occupation, people who the patient lived with, religion, referral source, reason for consultation at the ERU, diagnostic hypotheses recorded at the end of the attendance, whether clinical medications were prescribed, whether psychotropic medications were prescribed (and which ones, in affirmative cases), whether mechanical restraints were necessary, whether there was any dropout, referrals that were made, and prescriptions and procedures to be used at home. 

### Procedures

Every week from May 2010 to May 2011, the medical files of all patients who sought psychiatric consultations were separated and the data were transferred to a bookmarked collection, to build the database.

A data-gathering form was drawn up from the records of the ERU, and the variables of interest were included in it. A pilot study was conducted, consisting of a survey of 50 medical records from psychiatric care at the ERU, with the aim of adapting and standardizing the data gathering. The reasons for attendance reported at the beginning of the consultation were analyzed (noted by the resident in the "main complaint" field), and the diagnostic hypotheses were defined by the doctor at the end of the medical consultation. During the pilot phase, groupings of reasons and diagnostic hypotheses were defined, thus standardizing the data gathering to build the database. Among the reasons for medical care, situations such as memory problems, side effects of medications and prescription requests were classified as "others". Similarly, there were standardized categories relating to the referral source, prescriptions for medications and the referrals made. 

Throughout the data gathering, any doubts about the suitability of the raw data collected from the records of the ERU were discussed between the undergraduate student, a psychiatrist who was a member of the staff of the ERU, the first author of this study and the supervisor of the project.

The research project was approved by the Ethics Committee of the School of Medical Sciences, Unicamp (No. 269/2010).

## RESULTS

Between May 25, 2010, and May 31, 2011, 70,137 cases were attended, with 189 calls per day, on average. Of these, 1,835 (2.6%) were psychiatric consultations, corresponding to 1,465 patients, or 5 calls a day, on average. During these period, 238 patients were attended more than once by the psychiatric staff at the ERU. These cases were named repeaters and accounted for 16.2% of the cases cared for by the psychiatric staff. Table 1 shows the sociodemographic profile of the patients.

**Table 1:** Sociodemographic profile of patients seen at the psychiatric emergency unit

Characteristics	n	%
Gender		
Female	787	53.7
Male	678	46.2
Mean age	37.1 (± 13.9)	
Age group		
< 18 years old	66	4.5
18-24 years old	206	14.3
25-39 years old	602	41.8
40-59 years old	469	32.5
> 60 years old	97	6.7
Marital status (n = 990)		
Married/cohabiting	431	43.5
Single	383	38.6
Separated	146	14.7
Widowed	30	3.0
Race (n = 1356)		
White	1079	79.6
Brown	203	14.9
Black	71	5.2
Asian	3	0.2
Occupation (n = 797)		
Regular occupation	279	35.0
Unemployed	234	29.3
Retired	103	12.9
Housewife	73	9.1
Informal jobs	32	4.0
Student	76	9.5
Schooling (n = 759)		
≤ Middle school	326	42.9
High school	330	43.4
Post-secondary education	103	13.5
Living with (n = 765)		
Parents	316	41.3
Spouse + children	156	20.3
Alone	84	10.9
Only spouse	79	10.3
Only children	73	9.5
Other	57	7.4

Most patients (67%) originated from Campinas. The missing data rate was 1.7% for age, 32.4% for marital status, 7.5% for race, 50.7% for occupation, 48.1% for education level and 47.7% for cohabitation. Table 2 shows the referral source, the reasons for the consultation and the diagnostic hypotheses made at the end of the consultation. Table 3 shows data on the psychiatric management implemented during the attendance, namely: the pharmacological approach used, if any; whether there was any need for mechanical restraint; the dropout rate; prescriptions issued; and referrals on discharge.

**Table 2:** Clinical data of patients treated at the emergency psychiatric unit

Characteristics	n	%
Referral source		
Spontaneous	905	61.7
Other ERs*	188	12.8
Brought by family	98	6.6
SAMU†	70	4.7
Other services	70	4.7
Missing data	134	9.1
Reason for medical care		
Depressive symptoms	412	28.1
Agitation and behavioral change	346	23.6
Problems with alcohol and/or other drugs	286	19.5
Suicidal behavior	115	7.8
Ideas of persecution	93	6.3
Other	180	12.2
Missing data	33	2.2
Diagnostic hypothesis		
Disorders relating to use of PAS‡	338	23.0
Depressive disorder	272	18.5
Psychotic disorder	182	12.4
Anxiety/panic syndrome	132	9.0
Manic/hypomanic episodes	109	7.4
Adjustment reaction	68	4.6
Suicide attempt	66	4.5
Personality disorder	37	2.5
Other	200	13.6
Missing data	61	4.1

* ERs = Emergency Rooms

† SAMU = Mobile Emergency Attendance Service (Serviço de Atendimento Móvel de Urgência)

‡ PAS = psychoactive substance.

**Table 3:** Emergency referral unit management, prescriptions and referrals

Characteristics	n	%
Medication received at the ERU	339	23.1
Psychiatric medication received at the ERU	296	20.2
Medications prescribed at the ERU (n = 464)		
Benzodiazepine	218	46.9
Antipsychotic	145	31.2
Complex B/thiamine	38	8.1
Promethazine	48	10.3
Antidepressant	6	1.2
Biperiden	8	1.7
Mood stabilizer	1	0.2
Medications prescribed for home use (n = 959)		
Benzodiazepine	449	46.8
Antidepressant	222	23.1
Antipsychotic	218	22.7
Mood stabilizer	40	4.1
Complex B/thiamine	16	1.6
Biperiden	8	0.8
Promethazine	6	0.6
Medical restraint	69	4.9
Dropout	19	1.3
Referrals		
Primary public healthcare	374	31.6
Specialized clinics at HC	345	29.2
ASPA*	204	17.2
CAPS†	103	8.7
Psychiatric hospitalization	98	8.2
CAPS-AD	39	3.3
Inpatient care	11	0.9
Therapeutic community	7	0.5

ERU = emergency referral unit

HC = Hospital das Clínicas/Universidade Estadual de Campinas (Unicamp)

* ASPA = Psychoactive Substances Clinic (Ambulatório de Substâncias Psicoativas)

† CAPS = Psychosocial Care Center (Centro de Atenção Psicossocial)

**CAPS-AD:** = Psychosocial Care Center for alcohol and drug disorders (Centro de Atenção Psicossocial - Álcool e outras Drogas).

In comparing the repeaters (patients who sought psychiatric treatment more than once during the period) with patients who were treated only once, there were no significant differences between the two groups regarding gender, age, marital status, occupation, schooling level and main diagnostic hypotheses. The only significant difference between the groups was the reason for seeking attendance. Among the repeaters, the main reason was agitation/behavioral change (29.1%) and, in the group of non-repeaters, this reason was responsible for 23.1% of the visits (P = 0.05), and was the second largest cause of looking for help.

## DISCUSSION

Today, because of the clinical and social relevance of mental disorders, there is a need for studies that contribute towards the quality of medical care for this population, especially in clinical settings. It has been estimated that 25 to 30% of consultations with general practitioners are due to mental disorders.^9^ In this light, the present study was conducted to characterize a significant sample (n = 1,465) of patients who sought psychiatric care at an emergency service and discuss the clinical variables analyzed.

Regarding the patients' sociodemographic profile, this study showed that they were preponderantly female and Caucasian, with a mean age of 37 years, with slight predominance of married/cohabiting individuals, in regular employment, and particularly drew attention to the high rate of patients still living with their parents (41.3%), notwithstanding their age group. This profile is similar to what was described in other studies conducted in Brazil and elsewhere.^10^ ^,^ ^11^ However, it differed from two Brazilian studies^12^ ^,^ ^13^ performed in the cities of Ribeirão Preto and Sobral. These described a predominance of male patients, without conjugal bonds, with lower levels of education and professionally inactive. This discrepancy was probably due to the characteristics of each service, because in these places, a significant portion of the patients was treated because of alcohol withdrawal, which ended up being more compatible with the profile described. The present study included patients who directly sought psychiatric care. At the ERU of HC/Unicamp, the majority of alcohol withdrawal cases, especially involving *delirium tremens*, are primarily attended by other specialists, such as clinicians, surgeons, orthopedists and neurosurgeons, mainly because of other complaints that are consequent to or comorbid with alcohol dependence. Psychiatric care is often requested later on, to help deal with the mental and behavioral symptoms of these conditions. In the present study, interdisciplinary consultations and referrals for psychiatry were not analyzed.

Comparing the main reasons for seeking psychiatric emergency consultations with other studies conducted in Brazil and in other countries,^12^ ^,^ ^14^ ^,^ ^15^ most of the data showed similarities. However, in the present study, the rate of searching for psychiatric help due to attempted suicide was lower. This difference probably relates to the fact that at the ERU of HC/Unicamp, attempted suicide is generally primarily attended by medical clinicians, with support from the staff of the Poison Control Center (when the attempted suicide is due to substance overdose) or even from trauma surgeons (when it involves injury by firearms or knives) and the psychiatric staff is only brought in later on, for subsequent liaison consultations. It is important that non-psychiatric professionals and services involved in emergency care should receive training for recognizing, intervening and appropriately referring patients to psychiatrists, according to the main reasons for seeking help: symptoms of depression, psychomotor agitation/behavioral change and problems with alcohol and/or other drugs.^16^

Regarding the diagnostic hypotheses made by the physician at the end of the consultation at the ERU, psychoactive substance use disorders predominated, followed by depressive and psychotic disorders. This predominance of psychoactive substance use disorders has also been demonstrated in most other studies conducted in Brazil and internationally.^12^ ^,^ ^13^ ^,^ ^16^ ^-^ ^18^ A trend towards increasing numbers of emergency admissions for disorders relating to use of psychoactive substances has already been reported: in the United States, from 1995 to 2005, the rates increased from 0.65% to 3.7%.^19^ A national study conducted in the Psychiatric Emergency Unit of Hospital das Clínicas, Ribeirão Preto, São Paulo,^17^ which analyzed attendances from 1988 to 1997, showed that there was a progressive decrease in the proportion of non-psychotic disorders and an increase in the problems relating to alcohol and other drugs, schizophrenia and affective psychoses.

It needs to be borne in mind that establishing a psychiatric diagnosis requires longitudinal follow-up. In the emergency context, it is important to draw up diagnoses of the main psychiatric syndromes (descriptive definitions, with relatively constant clusters of certain signs and symptoms). These are primarily based on clinical presentation and on additional information relating to the background provided by the patient and accompanying persons.^16^ ^,^ ^20^

It is important to point out that there were differences in the descriptions of the situations that had motivated the search for assistance and in the hypotheses formulated by the professionals. While the presence of psychiatrists in emergency services is in itself exceptional in Brazilian hospitals, it is essential that physicians and nurses working in these services are able to recognize the conditions behind the complaints, especially to differentiate between possible causes of agitation and behavioral changes, which may indicate organic diseases, use of psychoactive substances or psychotic and manic syndromes, among other causes.^21^ Although the emergency context presents limitations on further and deeper diagnosis of psychiatric disorders, it is desirable for practitioners to be able to establish hypotheses and approaches that are as specific as possible. In this manner, emergency care can be the point of best qualification within the patients' healthcare network.^4^ ^,^ ^22^

Regarding pharmacological management at emergency units, the present study showed much lower rates of prescribing than in previous Brazilian studies.^12^ ^,^ ^13^ In one study,^12^ patients received medication in nearly 70% of the cases, compared with 23% in this study. This discrepancy may be due to the characteristics of each service. While most patients at the ERU of HC/Unicamp arise from spontaneous demand, the majority in the abovementioned study were referred from other services. Also, the psychiatric medical team at the ERU takes the view that the priority in emergency care is subsequent inclusion of the patient in the mental healthcare network. Thus, whenever possible, prescription of psychotropic medications is left for the professionals who will follow up the patient later on. Additionally, in the studies cited above, the services had exclusive spaces for patients to be cared for by psychiatric staff in the emergency unit, which may help patients to stay longer, such that they are more often treated at the service itself.

Regarding referrals made after the period of ERU care, our study showed that the primary care network was the main destination for referred patients, followed by the specialized psychiatric outpatient clinics of HC/Unicamp itself. This scenario is probably due to the characteristics of the population attended: predominance of spontaneous demand and of patients from Campinas favors referrals to the primary public mental healthcare network, and the presence of psychiatry residents in the service facilitates referral to the specialized psychiatric outpatient clinics of HC/Unicamp. Going against the recommendations of the Brazilian Ministry of Health, Psychosocial Care Centers (CAPS) were barely used as a referral alternative.^23^ The rate of referrals to psychiatric hospitalization (8.2%) was lower than reported in other Brazilian studies and internationally.^12^ ^,^ ^15^ ^,^ ^18^ This finding probably reflects the characteristics of the population, as well as the care network available in the city.

The most commonly prescribed medications at the time of patient discharge, in order of frequency, were benzodiazepines, followed by antidepressants and antipsychotics. This finding is similar to that of a study conducted in São Paulo,^10^ which indicated antidepressants, followed by benzodiazepines and antipsychotics. This highlighted the compatibility of the prescriptions with the leading diagnostic hypotheses formulated, namely, psychoactive substance use disorders and depressive and psychotic disorders.

Comparison of the repeater and non-repeater patients showed that the groups differed only with regard to the higher rate of psychomotor agitation/behavioral changes as a reason for searching for help, among the repeaters. A study conducted in Ribeirão Preto^12^ also showed no significant differences in sociodemographic profiles. However, we observed a significantly higher proportion of psychotic disorders and a lower proportion of substance use disorders among repeaters. It is possible that the number of repeaters limited the comparison, thus suggesting that studies with larger numbers of patients should be conducted in order to discuss other features that characterize repeater patients and thereby assisting in qualifying patient care measures.

It is important to take into consideration some limitations of this study. The first one relates to the incompleteness of the information in the medical records, notably sociodemographic variables. These variables were probably considered less essential than clinical data, which are fundamental for decision-making in emergency contexts. This difficulty has also been observed in other studies conducted in emergency services.^18^ ^,^ ^24^ ^,^ ^25^ This problem limits data gathering, thus resulting in high rates of missing data and hindering descriptive analyses and establishment of correlations.

Another limitation was the lack of standardization of diagnoses made in the ERU. Thus, standardization had to be established through *a posteriori* analysis of patients' medical files. Although this was after the pilot phase and through a consensus between three psychiatrists, there is no certainty of equivalence to the diagnosis that was made at the time when the care was provided.

A final limitation that deserves to be pointed out was the inclusion criterion that defined the study population as patients who sought psychiatric care, thus excluding consultations coming from referral from interdisciplinary consultations. This probably excluded from the study a considerable number of patients, with sociodemographic and clinical characteristics possibly differing from the population described, especially regarding the presence of clinical comorbidities and in cases of psychiatric care for children.

The limitations above indicate that caution should be used in extrapolating data to services with different characteristics.

Psychiatric care at emergency services constitutes an important link in the chain of care for these patients. Nonetheless, there is a scarcity of Brazilian studies characterizing both the population and the clinical factors involved. This is one of the largest national studies describing the population served by a psychiatric emergency team, and it also discusses the reasons for attendance and the management principles. The authors hope that this study has contributed towards planning actions designed to provide attention and care for patients seeking emergency services due to psychiatric conditions.

## CONCLUSIONS

The results from this study show that the profile of patients seen by psychiatric staff at the emergency unit consisted mostly of women, with an average age of 37 years, coming from spontaneous demand. The reasons for the consultations were mainly symptoms of depression, psychomotor agitation/behavioral changes or problems with psychoactive substances, and 16.2% of the patients had more than one psychiatric consultation at the service during the period (mainly due to psychomotor agitation/behavioral changes). The most frequent diagnostic hypotheses were psychoactive substance-related disorders and depressive and psychotic disorders. Less than a quarter of the patients were prescribed medication inside the service and almost half received prescriptions for home, especially benzodiazepines, antidepressants and antipsychotics. The patients were mainly referred to the primary mental healthcare network and to the university's specialized psychiatric outpatient clinics. The rate of hospitalizations was lower than in other reports in the literature.
